# Development and Characterization of New Pervaporation PVA Membranes for the Dehydration Using Bulk and Surface Modifications

**DOI:** 10.3390/polym10060571

**Published:** 2018-05-23

**Authors:** Maria Dmitrenko, Anastasia Penkova, Anna Kuzminova, Alexander Missyul, Sergey Ermakov, Denis Roizard

**Affiliations:** 1 St. Petersburg State University, 7/9 Universitetskaya Nab., St. Petersburg 199034, Russia; m.dmitrienko@spbu.ru (M.D.); ai.kuzminova@mail.ru (A.K.); s.ermakov@spbu.ru (S.E.); 2 ALBA Synchrotron Light Source, Carrer de la Llum 2-26, 08290 Cerdanyola del Vallès, Barcelona, Spain; amissiul@cells.es; 3 Laboratoire Réactions et Génie des Procédés, CNRS, Université de Lorraine, ENSIC, 1 rue Granville, 54000 Nancy, France; denis.roizard@univ-lorraine.fr

**Keywords:** polyvinyl alcohol, fullerenol, chitosan, layer-by-layer assembly, bulk modification, poly(allylamine hydrochloride), poly(sodium 4-styrenesulfonate), poly(acrylic acid)

## Abstract

In the present work, the novel dense and supported membranes based on polyvinyl alcohol (PVA) with improved transport properties were developed by bulk and surface modifications. Bulk modification included the blending of PVA with chitosan (CS) and the creation of a mixed-matrix membrane by introduction of fullerenol. This significantly altered the internal structure of PVA membrane, which led to an increase in permeability with high selectivity to water. Surface modification of the developed modified dense membranes, based on composites PVA-CS and PVA-fullerenol-CS, was performed through (i) making of a supported membrane with a thin selective composite layer and (ii) applying of the layer-by-layer assembly (LbL) method for coating of nano-sized polyelectrolyte (PEL) layers to increase the membrane productivity. The nature of polyelectrolyte type—(poly(allylamine hydrochloride) (PAH), poly(sodium 4-styrenesulfonate) (PSS), poly(acrylic acid) (PAA), CS), and number of PEL bilayers (2–10)—were studied. The structure of the composite membranes was investigated by FTIR, X-ray diffraction, and SEM. Transport properties were studied during the pervaporation separation of 80% isopropanol–20% water mixture. It was shown that supported membrane consisting of hybrid layer of PVA-fullerenol (5%)–chitosan (20%) with five polyelectrolyte bilayers (PSS, CS) deposited on it had the best transport properties.

## 1. Introduction

Polymers are widely used materials in different fields owing to their good mechanical and physicochemical properties as well as their economic accessibility [1,2,3]. The use of polymers for membrane production for applying in different membrane processes enables highly effective separation of liquid and gas mixtures. There are a large number of commercial polymer membranes that have undergone the necessary series of studies and found their application in membrane processes in industry due to their low cost, good film-formability, and mechanical strength. However, it should be noted that polymeric membranes are characterized by a number of drawbacks, such as low resistance to mechanical impurities, relatively poor chemical stability, low thermal resistance, and low permeability for some polymers. Thus, modern production conditions demand improved quality of the final product and productivity for existing processes. In this regard, the development of new membranes is a highly important task.

Among the membrane processes, pervaporation is one of the perspective membrane technologies that can be a good alternative to classical separation processes, applicable for liquid mixtures containing low-molecular weight components. It is widely used for the dehydration of water–organic mixtures, in particular containing an azeotropic and close-boiling components. One of the key factors for the effective performance of the pervaporation (high water content in the permeate and permeability) is the proper choice of a material type for membrane preparation [4,5,6].

Recently, a number of studies have been conducted using different types of hydrophilic polymers, especially poly(acrylic acid), cellulose, hydroxyl ethyl cellulose, chitosan (CS), sodium alginate, and poly(vinyl alcohol) (PVA) as membrane matrices for the pervaporation dehydration purposes [7,8]. Among these, PVA is considered to be one of the most widely used polymers in pervaporation, applied for the separation of water–organic mixtures, due to its unique properties such as good film-forming properties, high hydrophilicity, and chemical stability [9,10]. However, the permeability of PVA-based membranes is too low due to its glassy and semi-crystalline structure.

The transport characteristics of PVA membranes can be significantly improved using various methods of polymer modification and/or functionalization [9,11,12,13]. Among them, bulk (volume) and surface modifications are considered the most promising methods to get tailored membrane properties. In this regard, two modification methods were applied to PVA-based membranes in the present study.

Bulk modification was performed by the polymer blending (the addition of CS into PVA matrix) and the creation of a mixed-matrix membrane (by introducing fullerenol (polyhydroxylated fullerene) to PVA).

The blending of polymers is a quite simple bulk modification method capable to acquire characteristic advantages by combining two or more polymers in different concentration ratio for the preparation of the blended membrane with the optimal transport properties [14].

In this study, to increase the permeability of PVA membranes, the modification of PVA matrix by a well-known biopolymer chitosan (CS) was carried out. Chitosan was chosen owing to its high chemical resistance, biodegradability, nontoxicity, and high number of amino and hydroxyl groups, which actively react with PVA groups and significantly affect the changes in the membrane properties, depending on the polymer matrix content. The PVA-CS composite has been broadly reported and investigated for the application in various fields such as packaging in food industry [15], as hydrogels in medical and pharmaceutical industries [16], as films with a high antibacterial activity [17], and especially as a membrane material for gas separation [18], filtration [19], polymer electrolyte membrane electrochemical reactor (PEMER) [20] and pervaporation [21]. The literature review on the preparation of blended PVA-CS pervaporation membranes demonstrated that the studies focus on the dependence of the transport properties of the membrane on the weight ratio of both polymers in the membrane matrix. This, in turn, determines the prospects of using the membrane with a blended matrix for pervaporation of various mixtures. The developed blended PVA-CS membranes have been already tested for dehydration of 1,4-dioxane [22], tetrahydrofuran (THF) [23], ethylene glycol [24], ethanol and isopropanol [21,25], for separation of benzene/cyclohexane mixtures [26,27], binary and ternary mixtures of n-butyl acetate, n-butanol and water [28]. In the study [21], the main goals were to determine the optimal composition of the PVA-CS membranes, cross-linked by glutaraldehyde (GA) or by heat-treating at different temperatures, for the dehydration of isopropanol (IPA) by pervaporation (PV) and to investigate interactions among these variables. It was found that the CS content was the most important factor influencing the permeation flux and separation factor. PVA-CS blend membranes were also prepared by cross-linking of the PVA with urea formaldehyde/sulfuric acid (UFS) mixture for PV dehydration of isopropanol and tetrahydrofuran (THF) at 30 °C close to their azeotropic compositions in the study [23]. A new hollow fiber composite membrane of CS-PVA/polyvinylidene fluoride (PVDF) cross-linked with GA and sulfuric acid was prepared by casting the polymer solutions on PVDF hollow fiber support for PV dehydration of IPA [29]. The increase of thermostability and a decrease of hydrophilicity were shown after the blending CS and PVA by TGA and contact angles. The PVA and CS membranes, modified by the amino functionalized multi-walled carbon nanotube (NH_2_-MWCNT), were used for the dehydration of IPA, whilst the membrane with 10 wt % of NH_2_-MWCNT showed the optimal transport performance [30].

The creation of a mixed-matrix membrane is another promising method of modification, which allows changing the characteristics of the membrane material in a directed and flexible way, depending on the task solved. The main idea for the creating of this membrane type is the combination of the best properties and advantages of both components. These composite materials usually provide an effective integrated approach to solve practical problems, such as separation and recycling. Among the inorganic particles, carbon nanoparticles take a special place due to their physicochemical characteristics (for example, fullerenes [31], carbon nanotubes [32], graphene [33], graphene oxide [34], and fullerene derivatives [35], etc.). In this study, fullerenol as water-soluble fullerene derivative was chosen as an inorganic filler for the introduction into PVA-CS matrix, creating a mixed-matrix membrane (bulk modification), which already showed its promising applicability as a modifier and a cross-linking agent for the pervaporation PVA-based membranes in the previous works [36,37,38,39].

Among the various surface modification methods, the development of supported membranes with a thin selective layer and the deposition of nano-sized layers by layer-by-layer assembly (LbL) can be considered as effective means of adjusting of the membrane performance to obtain desired permeation flux and separation factor [40]. Membranes, that are covered by PEL layers on the surface, may have surface charge, and also have high hydrophilicity and, as a consequence, a strong affinity for water molecules, which makes polyelectrolytes attractive for their use in the manufacturing of the pervaporation membranes [40]. Depending on parameters—such as the amount of deposited layer, types of applied polyelectrolytes, their ionic strength, and pH—the structure and morphology of membranes can be varied in order to improve the efficiency of the pervaporation membranes [41,42,43,44]. In the article [44], a novel pervaporation LbL polyelectrolyte membrane with a single bilayer of polyethyleneimine (PEI) polycation and Nexar™ on a hydrolyzed polyacrylonitrile (PAN) hollow fiber substrate has been developed for the pervaporation dehydration of ethanol (85 wt %) with a good separation performance and long-term stability. The polyelectrolyte multilayer membrane, prepared by LbL adsorption of polyvinylamine (PVA) as cationic and polyvinylsulfonate (PVSu) as anionic component, was developed. It provided the improved transport properties for the separation of ethanol/water mixtures with low water content (<20 wt %), while the membrane modified by polyvinylsulfate (PVS) and polyacrylate (PAA) was suitable for the dehydration of feed with higher water content [45]. It was shown that this multilayer composite membrane exhibited improved transport parameters for the ethanol/water separation.

The increase of membrane permeability also can be reached by the creation of a supported membrane, consisting of a thin selective polymeric layer on the surface of a porous support allows to achieve a high permeability for commercial application. At present, supported membranes are used in various fields such as gas separation, water purification, pervaporation, etc. Dense polymeric membranes, which are very thin, usually do not possess sufficient mechanical properties and resistance, therefore, these should be deposited onto a stable support in order to avoid destruction and to create a thin selective layer to improve the performance. Various micro- and ultraporous commercial or prepared supports—consisting of mechanically rigid materials—are used for the development of a supported membrane with a thin selective PVA layer, depending on the separation task. Most of these materials are based on polysulfone and applied for copper ions removal and dehydration of ethanol mixtures [46,47]. Additionally, the folowing materials were used: α-Al_2_O_3_ hollow fiber or ceramic supports [48,49], poly(ether sulfone) (PES) [50,51], polypropylene [52], and polyacrylonitrile [53,54] for the pervaporation dehydration of various aqueous mixtures, etc. We used supported membranes based on composites PVA-CS and PVA-fullerenol-CS. These were developed by the deposition of a thin selective layer (~1 μm) onto the surface of the commercial ultrafiltration support (UPM-20) based on aromatic polysulfone amide. The choice of the UPM-20 support was based on an earlier study [55] showing good mechanical and chemical resistance of UPM-20, while other supports (including polyacrylonitrile (PAN)) could be hydrolyzed at elevated temperatures.

The obtained membranes modified in the bulk and on the surface with various methods were examined in separation of a model isopropanol (IPA)-water mixture to evaluate the changes of internal membrane structure and surface characteristics. Isopropanol is one the major industrial solvent and chemical intermediates for synthesis that is used in different fields such as chemical, pharmaceutical, medical, electronics, and cosmetic industries. Most frequently, absolutely dehydrated isopropanol is required. It is well-known that the dehydration of alcohols by traditional separation methods (distillation, rectification) is quite a difficult task due to the formation of azeotropic mixture with water (for example, the composition 12 wt % water–88 wt % IPA [56]). Energy demanding processes with special definite conditions and the use of harmful organic solvents that form stronger azeotropic mixtures with water, which prevents the recovery of a high-purity target product, are usually needed. Pervaporation with a properly matched membrane can be a promising way to dehydrate isopropanol owing to adequate separation efficiency, low energy consumption, and performance advantages without any additional reagents in comparison to traditional separation methods.

The aim of this study was to develop new PVA-based membranes having enhanced properties for the application to industrial dehydration of alcohol feed. To improve transport properties of PVA membranes, the different techniques for bulk and surface modifications were studied and implemented for the development of supported PVA composite membranes. Bulk modification included introduction of CS and fullerenol to PVA matrix. The choice of these modifiers was due to their nontoxicity, water-solubility, and biodegradability, which allows development of novel green membranes. Moreover, big amounts of highly reactive polar groups of such modifiers contribute to the increase of surface hydrophilicity and permeation flux of PVA-based membranes. The surface modification of obtained membranes based on composites PVA-CS and PVA-fullerenol-CS included the preparation of supported membranes on ultrafiltration commercial UPM-20 support and the deposition of PEL layers (by LbL) for the improved pervaporation performance of composite supported membranes. Moreover, the nature of water soluble PELs and deposited number of PELs were studied to find the optimal composition and conditions of surface modification. The transport properties of developed membranes were evaluated in separation of a model mixture of IPA (80 wt %) and water (20 wt %) by pervaporation. The stability and indelibility of PEL layers (PSS, CS) were studied by scanning electron microscopy (SEM) and measuring the contact angles by water.

## 2. Materials and Methods

### 2.1. Materials

Polyvinyl alcohol (PVA) with a molecular weight of 141 kDa from ZAO “LenReaktiv” (St. Petersburg, Russia) (Certificate of Analysis no. 553041-3013) was used as membrane material. The polyhydroxylated fullerene C_60_(OH)_12_ (Fullerene Technologies, Russia) was used as a bulk modifier and also as a cross-linking agent to create mixed-matrix membranes (MMMs). Chitosan (CS) from Sigma-Aldrich (St. Louis, MO, USA) was used as a bulk and surface modifier with medium molecular weight. Poly(sodium 4-styrenesulfonate) (PSS) and poly(acrylic acid) (PAA) were used as polyanions, while poly(allylamine hydrochloride) (PAH) and chitosan (CS) were used as polycations for surface modification by LbL assembly. Maleic acid (MA) with a purity of >99% was applied as an additional cross-linking agent for PVA membranes. Polyelectrolytes and MA purchased from Sigma-Aldrich (Lyon, France). Deionized water (MilliQ^®^ water) was used for all of the experiments and the preparation of polyelectrolyte and polymer solutions. Isopropyl alcohol (IPA), purchased from “Vekton” (Sankt-Peterburg, Russia), and acetic acid, from Sigma-Aldrich (Lyon, France), were used without additional treatment.

A porous support based on aromatic polysulfone amide (UPM-20, pore size 200 Å, from “Vladipor”, St. Petersburg, Russia) was chosen to develop the supported membranes with a thin top selective layer.

### 2.2. Methods of Membrane Preparation

#### 2.2.1. Composite Solutions

A 2 wt % aqueous solution of PVA was prepared by dissolving a predetermined sample of PVA powder in distilled water at 85 °C during 5 h with constant stirring. The chitosan solution (1 wt %) was prepared by dissolving chitosan powder in a 1 wt % aqueous solution of acetic acid with constant stirring during 4 h. Then the polymer solutions were filtered for removing any contaminants. The required amount of an aqueous solution of fullerenol (0, 1, 3, 5% *w*/*w* with respect to the polymer weight) was introduced into the solution of PVA with the following addition of chitosan (5, 10, 15, 20% *w*/*w* with respect to the PVA weight) and of maleic acid (35% *w*/*w* with respect to the PVA weight) to PVA composites in 24 h. Then it was dispersed by ultrasonic treatment with a frequency of 35 kHz for 40 min.

#### 2.2.2. Dense Membranes

For the preparation of dense membranes the required amount of polymer solution was poured on a Petri dish and after evaporation of the solvent and film formation all membranes were heat treated at 110 °C for 120 min for the chemical cross-linking.

#### 2.2.3. Supported Membranes

The supported membranes based on composites PVA-CS and PVA-fullerenol-CS with the addition of 35 wt % MA (the preparation composite method was described above) were prepared by casting of a thin selective layer of composite solution onto the surface of the commercial ultrafiltration support (UPM-20) followed by the drying at room temperature for 24 h for solvent evaporation and the formation of a thin layer. Also, these membranes were subjected to heat-treatment at 110 °C for 120 min for the chemical cross-linking [36,55].

The maximum content of chitosan and fullerenol in PVA matrix was limited to 20 and 5 wt %, respectively, because the rise of the concentration led to a deterioration of the mechanical properties of the membranes and to poor dispersion of nanoparticles in polymer solution. Maleic acid (35 wt %) was applied as the cross-linking agent in all membranes and the membranes were heated at 110 °C for 120 min. In Table 1 the designations of the developed membranes are presented in abbreviated form.

### 2.3. Methods

#### 2.3.1. Pervaporation Experiments

The pervaporation was performed using a laboratory cell in steady state mode with an effective membrane area of 15.2 cm^2^ at room temperature (22 °C) with constant stirring. The description of the pervaporation setup and the experimental conditions was presented in ref. [55]. The composition of the feed and permeate was determined by the gas chromatography method used SHIMADZU GC-201 (Shimadzu, Kyoto, Japan) chromatograph.

The membrane permeation flux J (kg/(m^2^h)) was calculated as the amount of liquid vaporized through a unit of the membrane area per hour and was calculated as (Equation (1)) [4]

J = W/(A∙t),(1)

where W (kg) is the weight of the liquids (water and isopropanol) that penetrated the membrane, A (m^2^) is the membrane area, and t (h) is the time of the measurement.

Each measurement was carried out at least three times to ensure a good accuracy of the transport parameters. The mean accuracy for the transport parameters were as follows: for dense membranes, ±0.2% for water content in the permeate and ±2% for permeation flux; for supported membranes, ±0.1% for water content in the permeate and ±1% for permeation flux.

#### 2.3.2. IR Spectroscopy

The spectra were recorded in the range 680–4000 cm^−1^ at 25 °C with a resolution of 0.5 cm^−1^ on an Fourier transform infrared spectrophotometer IRAffinity-1, Shimadzu, Kyoto, Japan.

#### 2.3.3. X-ray Diffraction Analysis

The structure of the membranes were investigated at room temperature by Bruker D8 DISCOVER diffractometer (Karlsruhe, Germany) (40 kV, CuK_α_ radiation, step size 0.05°, 40 mA, scan rate 5 s/step) for 2θ = 5 ÷ 70° in the Bragg–Brentano geometry. The SAXS data were analyzed using ATSAS software package [57].

#### 2.3.4. Scanning Electron Microscopy

SEM micrographs of the cross-sections of dense and supported membranes were obtained by a Zeiss Merlin SEM. The dense and supported membranes were submerged in liquid nitrogen and fractured perpendicular to the surface. The prepared samples were observed using SEM at 1 kV.

#### 2.3.5. Contact Angle Determination

Contact angles were measured as described in the study [58]. For dense membranes, measurements were taken from both sides of the membrane, while for supported membranes it was done only from the selective PVA layer to study the hydrophilicity/hydrophobicity of the membrane surface.

#### 2.3.6. Swelling Experiment

The swelling degree of the dense polymer membranes was studied by gravimetric method. The membranes of known weight were immersed in a weighing bottle with water (a component of a separated mixture in pervaporation). The membranes were weighed daily until a constant weight value. The degree of equilibrium swelling S_0_ (%) was calculated using the equation

S_0_ = [(m_n_ − m_0_)/m_0_]∙100%, (2)

where m_n_ is the weight of the swollen membrane, m_0_ is the initial weight of the dry sample.

#### 2.3.7. Layer-by-Layer Deposition Technique

PAH, PSS, PAA of the concentration (10^−2^ mol/L) and chitosan (4.7 wt % in 1 wt % acetic acid solution) were used as polyelectrolyte solutions.

The deposition of nano-sized polyelectrolyte layers was carried out by the ND multi-axis dip coater ND-3D 11/5 robot. The membrane was clamped and immersed in polyelectrolyte solution for 10 min. The polyanion (PSS or PAA) solution was deposited first, and then the membrane was removed and thoroughly rinsed with water. After the membrane was immersed in the polycation (PAH or CS) solution for 10 min, the same water rinsing process of the membrane was repeated. Thus, one bilayer of polyelectrolytes was formed on the surface of the membrane. Similarly, additional bilayers were deposited until the required number of bilayers was reached. The numbers of deposited polyelectrolytes bilayers were varied from 2 to 10 on the surface of a supported membrane.

The charge concentration *ρ_c_* was calculated as the ratio of the number of ion pairs (=1) to the number of carbon atoms per repeat unit of the cationic and anionic polyelectrolytes [41], e.g., for PSS/PAH *ρ_c_* is 1/(8 + 3) = 0.09, for PSS/CS *ρ_c_* is 1/(8 + 8) = 0.0625, for PAA/CS *ρ_c_* is 1/(3 + 8) = 0.09.

## 3. Results and Discussion

Currently, eco-friendly polymeric membranes with improved transport properties are required for the dehydration of water–organic mixtures by pervaporation. At the moment, the research is directed to the development of supported membranes, which would be promising for industrial purposes, using several methods of functionalization. Thus, in the present work, chemically cross-linked dense and supported membranes based on PVA have been developed with the use of bulk (volume) and surface modifications. At first, dense membranes based on PVA were subjected to the bulk modification using various approaches such as the blending of PVA with chitosan (CS) and the creation of mixed-matrix membranes by the introduction of fullerenol into the polymer matrix. These dense membranes based on composites PVA-chitosan and PVA–fullerenol–chitosan were created for the investigation of the modifier impact on the transport characteristics of PVA-based membranes, due to the fact that in the study of dense membranes, the influence of the support and defects of the selective layer can be excluded. To improve transport properties by reducing the thickness of the hybrid membranes, it was decided to develop a supported membrane with a thin selective layer based on PVA composites casting on a commercial ultrafiltration support UPM-20, which did not affect the mass transfer of the components through the membrane (1st possibility of the surface modification). The chemically cross-linked supported membranes with a selective layer based on PVA-chitosan and PVA–fullerenol–chitosan nanocomposites were subjected also to the second way of surface modification by LbL deposition of polyelectrolytes to improve performance. The effect of the bulk (the introduction of fullerenol and chitosan into the PVA matrix) and the surface (development of the supported membranes and application of LbL assembly for deposition of PEL nano-layers such as poly(allylamine hydrochloride) (PAH), poly(sodium 4-styrenesulfonate) (PSS), poly(acrylic acid) (PAA), and chitosan (CS)) modifications on the transport properties of PVA membranes was studied during the separation of the isopropyl alcohol–water mixture by pervaporation.

### 3.1. Bulk Modification Methods

#### 3.1.1. Modification by Polymer Blends

**Figure 1:**
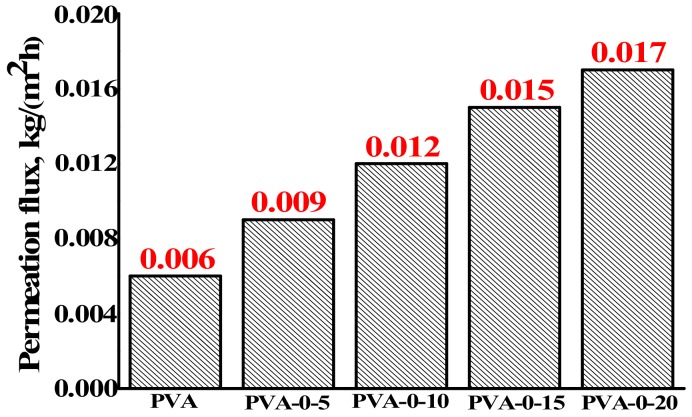
The permeation flux for the dense membranes for the separation of 80 wt % isopropanol–20 wt % water mixture by pervaporation at 22 °C. The accuracy value for permeation flux did not exceed ±2%.

Blends of PVA with chitosan in different proportion of weight fraction (5, 10, 15, and 20 wt % of CS to PVA) has been performed to average the properties of these two polymers and improve the performance of the PVA membranes. Transport properties of the developed dense membranes, based on the PVA-CS composites, were investigated for the separation of isopropanol (80 wt %)–water (20 wt %) mixture by pervaporation at 22 °C. All membranes were highly selective to water (the water content in the permeate was 99.9 wt %). The dependence of permeation flux for dense membranes on the CS concentration in PVA matrix is presented in Figure 1.

As can be seen from the data in Figure 1, the increase of chitosan concentration into the PVA matrix led to an increase of the permeation flux, while the selectivity remained constant (99.9 wt % water content in the permeate). This fact may be related to a decrease in the crystallinity of the PVA-chitosan membrane with the increase of CS in the matrix because of the interaction between CS and PVA groups (–NH_2_ and –OH) [21]. A membrane containing 20 wt % chitosan had the largest permeation flux and was chosen for further modification by fullerenol for the development of mixed-matrix (hybrid) membranes.

#### 3.1.2. Creation of Mixed-Matrix Membranes

**Figure 2:**
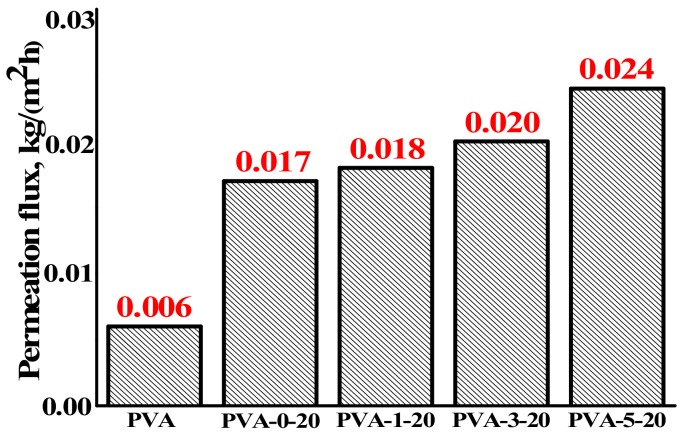
The permeation flux for the dense membranes with fullerenol for the separation of 80 wt % isopropanol–20 wt % water mixture by pervaporation at 22 °C. The accuracy value for permeation flux did not exceed ±2%.

Different concentrations of fullerenol (1–5 wt % to PVA) were introduced into PVA-chitosan blend matrix for increasing the performance of the membrane. The choice of this nanoparticle is justified due to the previous studies, which showed the promising modification of PVA membranes by fullerenol, improving transport properties for the pervaporation dehydration of water–organic mixtures [36,37,38,39,55]. The transport properties of the developed hybrid membranes based on PVA–fullerenol–chitosan composites were also examined for the separation of 80 wt % isopropanol–20 wt % water mixture by pervaporation at 22 °C. The high selective properties with respect to water, i.e., 99.9 wt % in the permeate, for all modified membranes were noticed. The dependence of permeation flux for these membranes on the fullerenol content in PVA-CS (20 wt %) matrix is presented in Figure 2.

The introduction of fullerenol into the PVA-CS (20 wt %) blend matrix up to 1 wt % led to similar values in the permeation flux if compared to PVA-CS (20 wt %) membrane (Figure 2) because the small content of fullerenol in PVA-CS membrane did not provide the significant difference in the permeation flux (the permeation flux for PVA membrane was represented in Figure 2 for the comparison to membranes modified by fullerenol). However, the PVA-5-20 membrane with 5 wt % fullerenol and 20 wt % chitosan demonstrated the best transport properties (permeation flux 0.024 kg/(m^2^h), 99.9 wt % water in the permeate) for the pervaporation dehydration of isopropanol. This effect occurred due to the presence of hydroxyl groups of fullerenol that led to the significant hydrophilization of the membrane surface (Section 3.2.4, Table 3).

### 3.2. Structure Characteristics of the PVA–Fullerenol–Chitosan Membranes

The structure of the membranes based on PVA–fullerenol–chitosan and PVA–chitosan composites was studied by FTIR spectroscopy and X-ray diffraction to confirm the interaction of PVA with fullerenol and/or chitosan. The internal structure and the surface of the studied membranes were additionally investigated and evaluated by scanning electron microscopy (SEM), contact angle measurements, and sorption experiments.

#### 3.2.1. Study of the Membrane Structure by FTIR Spectroscopy

**Figure 3:**
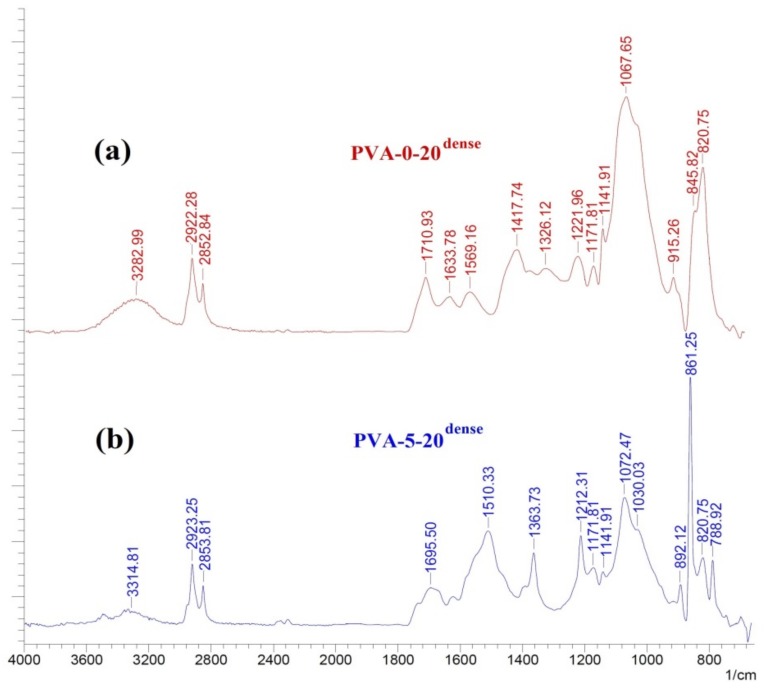
IR spectra of (**a**) PVA-0-20^dense^; (**b**) PVA-5-20^dense^ samples.

FTIR spectroscopy was applied to study the nature of interaction between the components of the hybrid membrane (PVA, fullerenol, chitosan). It should be mentioned that all membranes additionally contained maleic acid (35 wt %) as a cross-linking agent. The FTIR spectra for PVA-0-20^dense^ and PVA-5-20^dense^ membranes are presented in Figure 3.

The data demonstrated that there was a wide peak in the region 3200–3500 cm^−1^ for PVA-0-20^dense^ membrane, which corresponded to the oscillations of –OH groups of PVA (Figure 3a). While for PVA-5-20^dense^ membrane the intensity of a given peak decreased due to the interaction between hydroxyl groups of PVA and fullerenol (Figure 3b) [36] with the formation of ether bonds as well as ester bonds, related to the interaction of PVA and MA, and also cross-linking between MA and fullerenol. The peaks observed between 2840 and 3000 cm^−1^ referred to the asymmetric and symmetrical stretching of -CH_2_ groups. The peak, situated at 1171.81 cm^−1^ on both spectra (Figure 3), characterized the saccharide group of chitosan, whereas the peaks in the region 1570–1655 cm^−1^ indicated the presence of amides in the composites.

#### 3.2.2. Small-Angle X-ray Diffraction

**Figure 4:**
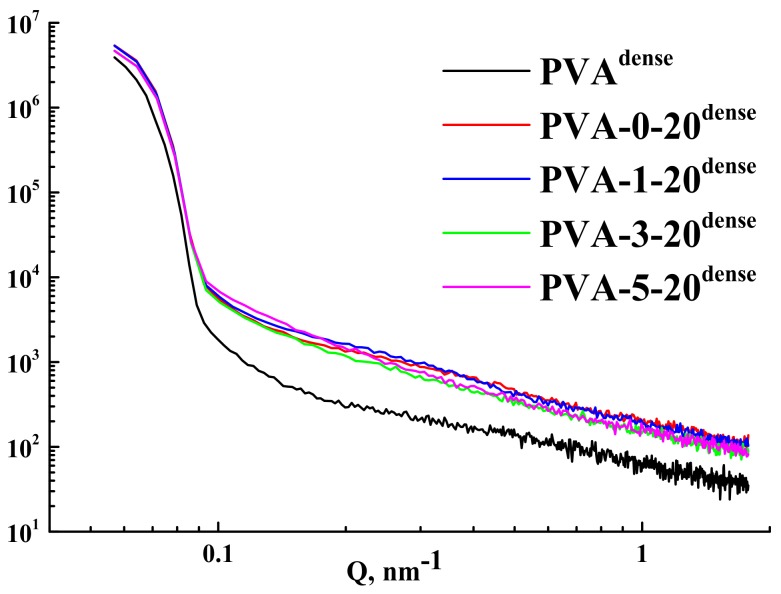
SAXS patterns of the membranes.

The structure of the modified membranes was characterized by SAXS. The obtained SAXS patterns are presented in Figure 4.

The calculated results from SAXS data are presented in Table 2.

It was shown that the radius of gyration and Porod volume slightly increased when chitosan was added to the initial polymer, reflecting higher degree of cross-linking in accordance with swelling degree data. This change persists after addition of up to 3 wt % of fullerenol. However, the introduction of 5 wt % fullerenol in PVA-chitosan matrix causes the decrease of both parameters with a drastic fall, due to the significant change of the PVA microstructure (Table 2). The Porod volume of the polymer globules corresponds to the molecular weight of the globule being approximately four times larger than the average molecular weight of the polymer before this transition while the addition of 5 wt % fullerenol leads to the globules of smaller average diameter (~three molecular weight of PVA). The obtained rearrangement of the microstructure for PVA-5-20^dense^ has a significant effect on the physicochemical and transport properties that are presented in the further sections.

#### 3.2.3. SEM Analysis of Dense Membranes

**Figure 5:**
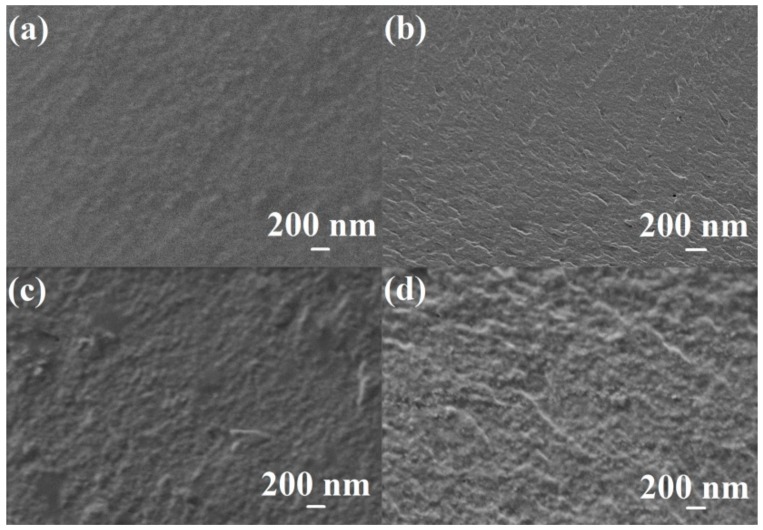
Cross-sectional SEM micrographs of (**a**) PVA-0-20^dense^; (**b**) PVA-1-20^dense^; (**c**) PVA-3-20^dense^; and (**d**) PVA-5-20^dense^ membranes (200 nm).

A detailed study of the inner structure of the dense membranes was carried out by scanning electron microscopy (SEM). Figure 5 demonstrates the cross-sections of the dense membranes based on PVA-chitosan (20 wt %) with the introduction of 1–5 wt % fullerenol.

The presented SEM micrograph (Figure 5a) demonstrates that chitosan is uniformly dispersed in the PVA matrix without visible clusters and voids in the membrane film, which indicates the absence of defects. However, an increase of fullerenol concentration in PVA-chitosan (20 wt %) matrix (from 1 to 5 wt %) leads to an increase in the roughness of the cross-section surfaces of the membranes (Figure 5b–d).

#### 3.2.4. Contact Angle Measurements

The study of the changes in the surface morphology of the dense membranes based on PVA, PVA–chitosan, and PVA–chitosan–fullerenol composites was carried out by the static sessile drop method to measure the contact angles by water. The water was chosen as a standard liquid to determine the hydrophilicity of the membrane surfaces. The values of the obtained contact angles for the dense membranes after the bulk modifications with chitosan and fullerenol are presented in Table 3.

The presented data in Table 3 demonstrated that the introduction of 20 wt % chitosan in the PVA matrix did not lead to a change in the hydrophilic surface properties of the membranes, while increasing of fullerenol concentration into the PVA-chitosan matrix led to a decrease of the contact angle, namely to the surface hydrophilization, due to the rise of the quantity of polar groups (–OH) on the surface of the membrane. This phenomenon significantly affects the transport properties of fullerenol-containing membranes.

#### 3.2.5. Study of the Swelling Degree

The degree of swelling in water has been studied for the developed dense membranes based on PVA, PVA–chitosan, and PVA–fullerenol–chitosan composites by the gravimetric method. The swelling characteristics were studied to assess the cross-linking degree of PVA membranes after carrying out bulk modifications as well as to study the degree of swelling in water, since the first stage of the pervaporation mechanism is sorption. The values of the swelling degree are necessary for understanding the transport mechanism of low-molecular penetrants through the membrane. Table 4 presents the data of swelling degree in water for the dense PVA-based membranes.

As it can be seen from the data given in Table 4, the introduction of chitosan into the PVA matrix contributes to a slight decrease in the swelling degree of the membrane in water, which means that the polymer chains are not further cross-linked. The introduction of 1 and 3 wt % of fullerenol into the blending polymer matrix also did not lead to a significant change in the swelling degree, while an increase in the fullerenol concentration up to 5 wt % in the membrane matrix led to a significant decrease in the swelling degree due to the stronger cross-linking degree of the polymer chains by hydroxyl groups of fullerenol [36].

### 3.3. Surface Modification Methods

#### 3.3.1. Development of the Supported Membranes

**Figure 6:**
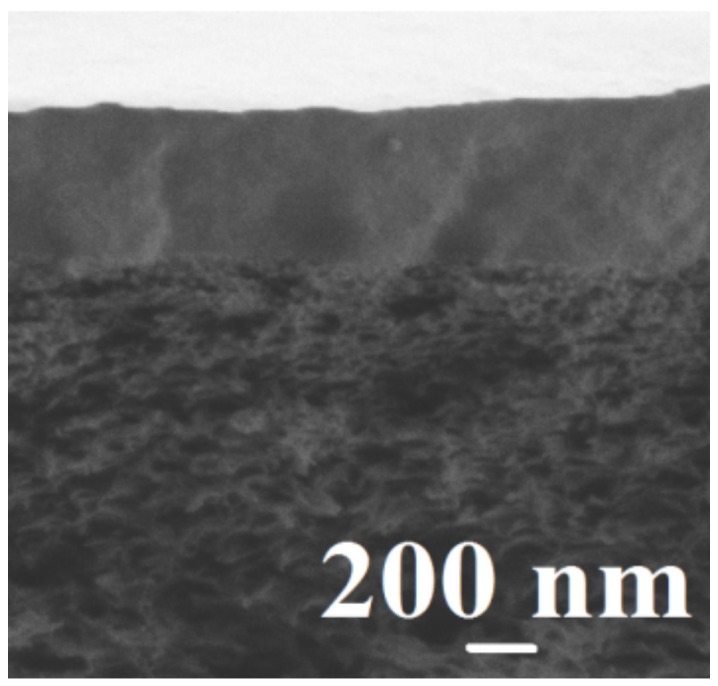
SEM micrograph of the cross-section of the supported PVA-5-20 membrane.

To further increase the performance of the membranes for the prospective application in industry, the modification of membrane surface was performed by the development of supported membranes consisting of a thin selective layer based on PVA-0-20 and PVA-5-20 deposited onto the ultrafiltration commercial polymer support UPM-20 (Figure 6).

Two areas can be distinguished on a micrograph: the region of a porous support and a thin selective non-porous layer with 1 ± 0.3 μm thickness. The presented micrograph demonstrates a good adhesion of the selective layer to the polymer support, as well as a uniform distribution of the selective layer on the support surface without penetration of the polymer solution into the pores.

The transport properties of these supported membranes, as in case of dense membranes, were investigated during the separation of 80 wt % isopropanol–20 wt % water mixture by pervaporation at 22 °C. The obtained results are presented in Table 5.

The data presented in Table 5 demonstrate that the permeation flux of supported membranes is ca. 10 times larger than for the dense membranes (Section 3.1.2, Figure 2). The fullerenol-containing membrane PVA-5-20 has higher permeation flux and water content in the permeate if compared to PVA-0-20 membrane. The improvement in the permeability can occur due to an increase of the number of hydroxyl groups on the surface of the membrane, modified by fullerenol and, consequently, an increase in the number of transport channels for water transfer during the dehydration of isopropanol. The result is in agreement with the data of SEM and the contact angles (Figure 5, Table 3). The improvement of the water content in the permeate of PVA-5-20 membrane is due to a deeper cross-linking of the polymer chains, which is in agreement with the swelling data (Section 3.2.5, Table 4).

#### 3.3.2. LbL Assembly

##### Modification by PSS as Polyanion and PAH or CS as Polycations

It should be noted that it is very important to control membrane surface characteristics, which play important roles in the first stage of pervaporation mass-transfer mechanism (sorption of components). To increase the membrane performance the deposition LbL method of PEL nano-sized layers was applied for the surface modification of hybrid supported membranes. Poly(sodium 4-styrenesulfonate) (PSS) was chosen as a widely used strong polyanions, and poly(allylamine hydrochloride) (PAH) and chitosan (CS) were used as the polycations, which were chosen due to their good adhesion properties and optimal charge density [41]. To optimize the surface modification of supported PVA membranes, 5 and 10 bilayers of the following PEL pairs (PSS and PAH, PSS and CS) were deposited on the membrane surface. To compare membrane transport properties the developed membranes were also tested in pervaporation of 80 wt % isopropanol–20 wt % water mixture at 22 °C (Table 6).

The presented data demonstrate that the LbL modification led to an increase in permeation flux and a decrease in water content in the permeate compared to the unmodified by LbL membranes (Table 5) that could be caused by the formation of small hydrophilic mashes in PEL layers due to the high charge PEL density. This led to less tight structure and the increasing the hydrophilicity of the membrane surface, thereby providing an increased permeability with reduced water content in the permeate [41]. Also, this fact could be caused by intrinsic charge compensation occurred in the PEL bulk, which determined the membrane transport properties [40]. The application of the pair PSS and PAH for LbL modification resulted in lower permeability of the membranes, compared to the PSS and CS modified membranes, due to the higher charge concentration (0.09 as compared to 0.0625 for PSS/CS) [41] and a denser electrostatically PEL cross-linking [40]. The data in Table 6 demonstrate that the most effective LbL modification was achieved by casting five bilayers of polyelectrolytes PSS and CS, which led to the increase of permeation flux in ca. 1.4 times with an equal content of water in the permeate compared to PVA-0-20 and PVA-5-20 membranes (Table 5). However, the deposition of 10 bilayers of PSS and CS on the PVA-0-20 and PVA-5-20 membrane surfaces led to the decrease of permeation flux because the increase in the membrane thickness inhibited the diffusion of penetrants in PEL layer to the selective PVA layer. Additionally, there was a decrease of water content in the permeate, due to the facilitated water sorption induced by the formation of bigger amount of the polar mashes in PEL layer and the increase of surface hydrophilicity, caused the simultaneous i-PrOH penetration with water. The PVA-5-20/LbL-5^PSS,CS^ membrane possessed the best transport properties for the dehydration of isopropanol (20 wt % water) through the pervaporation: permeation flux 0.34 kg/(m^2^h), 95.6 wt % water in the permeate.

To study the effect of varying the number of bilayers on the transport properties of the best PVA-5-20/LbL-5^PSS,CS^ membrane, it was decided to develop membranes with two and seven bilayers of PSS and CS, the obtained transport data after pervaporation of 80 wt % isopropanol–20 wt % water mixture at 22 °C are presented in Table 7.

It was demonstrated that the deposition of two PEL bilayers on the membrane surface is not sufficient to create uniform coating without defects on the PVA-5-20 membrane. The reason is, the transport characteristics for PVA-5-20/LbL-2^PSS,CS^ membrane for the dehydration of isopropanol (20 wt % water) by the pervaporation at 22 °C are comparable to the properties of the unmodified PVA-5-20 membrane (Table 5). However, the increase of PEL bilayer numbers from two to five led to an ensured increase in permeation flux with a slight decrease in water content in the permeate of the membranes. This is confirmed by the formation of continuous PEL layer without any defects on the membrane surface (Figure 6) with highly polar mashes, which improved the permeability of polar water molecules compared to less polar isopropanol. While there is no change in permeation flux for the deposition on the membrane surface of seven PEL bilayers and a significant decrease in selectivity was noticed (93.1 wt % water content in the permeate for PVA-5-20/LbL-7^PSS,CS^ membrane). This is explained by increasing the number of mashes in PEL layer and the hydrophilicity of the surface. It was shown that PVA-5-20/LbL-5^PSS,CS^ membrane with five bilayers of PSS and CS on the surface had the best and optimal transport properties for isopropanol dehydration.

##### Variation of LbL Modification Conditions for the PVA-5-20/LbL-5^PSS,CS^ Membrane

The transport properties after the pervaporation of 80 wt % isopropanol–20 wt % water mixture at 22 °C for supported PVA-5-20 membrane with a variation of LbL modification conditions are presented in Table 8.

The attempts to change the final (upper) CS layer to a PSS layer on the membrane surface by developing a membrane, based on the PVA-5-20 with 5.5 bilayers to study the impact of upper PEL layer on pervaporation separation did not bring significant improvements and changes (Table 8). For the pervaporation separation of isopropanol–water (80/20 wt %) mixture using the obtained membrane with 5.5 PEL bilayers, a ca. 1.2 times decrease of the permeation flux (0.291 kg/(m^2^h)) with comparable selectivity (95.6 wt % water in the permeate) comparing to the PVA-5-20/LbL-5^PSS,CS^ membrane (Table 6) was noticed, which indicated the futility of using PSS polyelectrolyte as the final layer on the membrane surface.

Moreover, to study the nature of polyelectrolytes, deposited on the surface of a selective PVA layer, in the process of the preparation PVA-5-20/LbL-5^PSS,CS^ membrane PSS polyelectrolyte as polyanion was replaced with another one such as polyacrylic acid (PAA) (Table 8). This membrane, based on PVA-5-20 composite and modified by five bilayers of PAA and CS, had a permeation flux of 0.267 kg/(m^2^h) and 89.6 wt % water in the permeate, during the separation of the same content of isopropanol–water mixture (80 wt % isopropanol–20 wt % water) by the pervaporation. The obtained value for the permeation flux is lower if compare to the PVA-5-20 membrane, modified by five bilayers of polyelectrolites PSS and CS (0.340 kg/(m^2^h)) (Table 7). This is in accordance with a dependence of the charge density on permeation flux. It is known that these parameters are changing in reversed order. The charge concentration for PAA and CS pair is equal to 0.09.

It should be mentioned that in the already published paper [11] it was reported that the optimal number for PSS/PAH polyelectrolyte pair on the surface of PVA–fullerenol–PAH supported membrane to improve membrane properties for isopropanol dehydration (permeation flux 0.286 kg/(m^2^h) and water content in the permeate 98.4 wt % for pervaporation of 80 wt % isopropanol–20 wt % water mixture at 20 °C) was 10. While in the present study it was shown that the change of PAH to CS, during bulk modification and the additional replacement of the PAH to CS during surface modification, led to the 1.2 times increase of the permeation flux, if compared with the developed PVA-fullerenol (5%)-PAH/LbL-10 membrane, and 10 times, if compared with the commercial analogue PERVAP^TM^ 1201 [11].

Thus, correctly selected polyanion–polycation pair and their deposition order by LbL equipment, as well as bulk modifiers (chitosan and fullerenol), have a significant effect on the transport characteristics of the membranes. These pervaporation experiments present once again that the membrane PVA-5-20/LbL-5^PSS,CS^ with five bilayers of PSS and CS on the surface has the best and optimal transport properties.

##### Study of the PEL Layer Stability for PVA-5-20/LbL-5^PSS,CS^ Membrane

The contact angles and the cross-section of the best PVA-5-20/LbL-5^PSS,CS^ membrane were investigated before and after the pervaporation by the static sessile drop method and SEM, respectively, and, additionally, the membrane was immersed in bidistilled water for four days. These experiments were carried out to evaluate the stability of the PEL layer on the surface of the PVA-5-20/LbL-5^PSS,CS^ membrane.

It was shown that the contact angle for this membrane did not change, it had the same values before (79 ± 2°) and after the pervaporation (77 ± 2°) of isopropanol–water (80/20 wt %) mixture at 22 °C.

**Figure 7:**
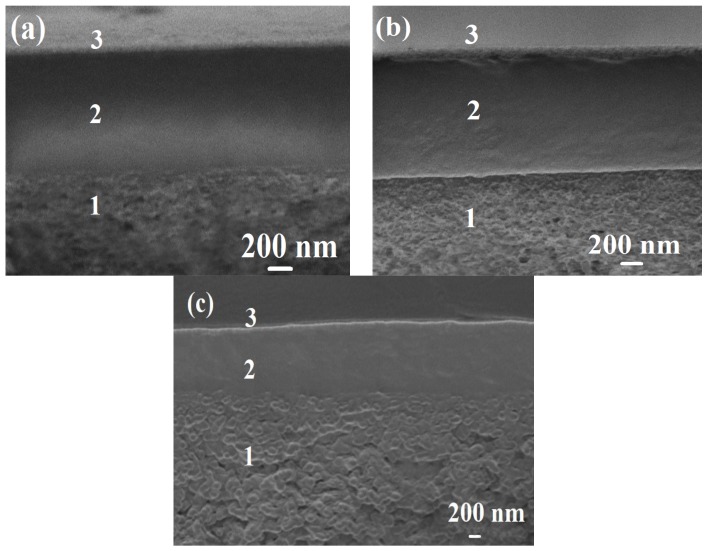
SEM micrographs of cross-section for the supported PVA-5-20/LbL-5^PSS,CS^ membrane (**a**) before and (**b**) after the pervaporation; (**c**) after membrane immersion in water for four days.

SEM micrographs, presented in Figure 7, demonstrate the cross-section of PVA-5-20/LbL-5^PSS,CS^ membrane before (a) and after (b) the pervaporation experiment, (c) after membrane immersion in water for four days.

Three areas can be clearly distinguished in the SEM micrographs (Figure 7): (1) the region of the porous support, (2) the selective layer (1 ± 0.3 μm), and (3) the polyelectrolyte layer of PSS and CS (0.4 μm). Notably, the thickness of the layers remained unchanged and continuous throughout the membrane surface after pervaporation.

The SEM and contact angle data show that the surface of the membrane did not change, which indicated the polyelectrolyte layers to be stable on the membrane surface and not to be washed away, enabling the application of this PVA-5-20/LbL-5^PSS,CS^ membrane with improved transport properties for industrial purposes of dehydration in the future.

## 4. Conclusions

In this study, the novel dense and supported mixed-matrix membranes were developed by the use of bulk (introduction of chitosan and fullerenol to PVA matrix) and surface (LbL techniques with the application of different PEL pairs and bilayer numbers) modification methods.

It was shown that application of the bulk modification by the addition of up to 20 wt % chitosan to PVA dense membranes led to the 2.8 times increase in the permeation flux if compared to unmodified PVA membrane with high water content in the permeate (99.9%) during the dehydration of 80 wt % isopropanol, due to the reduced crystallinity of the PVA/chitosan blend membrane. Additional introduction of 5 wt % fullerenol to PVA/chitosan blend membrane causes even greater increase in the permeation flux (four times). This effect occurs due to the presence of hydroxyl groups of fullerenol that cause significant hydrophilization of the membrane surface, that was confirmed by contact angle measurements.

The development of the novel supported PVA based membranes and the use of surface modification for them through applying of LbL assembly significantly improve the transport properties of the membranes. It was shown that a correctly selected polyanion–polycation pair and their deposition order by LbL equipment, as well as bulk modifiers (chitosan and fullerenol), have a significant effect on the transport characteristics of the membranes. Among the used polyelectrolyte pairs (PSS/CS, PSS/PAH, PAA/CS), the best transport properties for the isopropanol dehydration were noticed for PVA membrane, containing 20 wt % chitosan and 5% fullerenol with additional modification by five bilayers of PSS/CS (PVA-5-20/LbL-5^PSS,CS^): permeation flux 0.340 kg/(m^2^h) and water content in permeate 95.6 wt % for the separation of 80 wt % isopropanol–20 wt % water mixture at 22 °C. This could be explained by the intrinsic charge compensation and optimal charge concentration of this polyelectrolyte pairs (0.0625) if compared to PSS/PAH and PAA/CS.

## Figures and Tables

**Table 1:** Prepared PVA membrane samples.

Membranes	Type	Thickness, µm	Bulk Modification		Surface Modification	
			Fullerenol wt %	Chitosan wt %	Quantity of Bilayers	Type of PELs
PVA^dense^	dense	40	-	0	-	-
PVA-0 *-5 *^dense^	dense	40	-	5	-	-
PVA-0-10^dense^	dense	40	-	10	-	-
PVA-0-15^dense^	dense	40	-	15	-	-
PVA-0-20^dense^	dense	40	-	20	-	-
PVA-1-20^dense^	dense	40	1	20	-	-
PVA-3-20^dense^	dense	40	3	20	-	-
PVA-5-20^dense^	dense	40	5	20	-	-
PVA-0-20	supported	1	-	20	-	-
PVA-5-20	supported	1	5	20	-	-
PVA-0-20/LbL-5^PSS,PAH^	supported	1	-	20	5	PSS,PAH
PVA-0-20/LbL-10^PSS,PAH^	supported	1	-	20	10	PSS,PAH
PVA-0-20/LbL-5^PSS,CS^	supported	1	-	20	5	PSS,CS
PVA-0-20/LbL-10^PSS,CS^	supported	1	-	20	10	PSS,CS
PVA-5-20/LbL-5^PSS,PAH^	supported	1	5	20	5	PSS,PAH
PVA-5-20/LbL-10^PSS,PAH^	supported	1	5	20	10	PSS,PAH
PVA-5-20/LbL-5^PSS,CS^	supported	1	5	20	5	PSS,CS
PVA-5-20/LbL-10^PSS,CS^	supported	1	5	20	10	PSS,CS
PVA-5-20/LbL-2^PSS,CS^	supported	1	5	20	2	PSS,CS
PVA-5-20/LbL-7^PSS,CS^	supported	1	5	20	7	PSS,CS
PVA-5-20/LbL-5.5^PSS,CS^	supported	1	5	20	5.5	PSS,CS
PVA-5-20/LbL-5^PAA,CS^	supported	1	5	20	5	PAA,CS

* To simplify the designation of membranes, the first number after PVA refers to the content of fullerenol wt %, the second—to the content of chitosan wt %.

**Table 2:** Calculated characteristics from SAXS data.

Membrane	RG, nm	Porod Volume, 10^5^ nm^3^
PVA^dense^	46.4	9.13
PVA-0-20^dense^	46.7	9.43
PVA-1-20^dense^	47.1	9.63
PVA-3-20^dense^	46.8	9.41
PVA-5-20^dense^	43.8	7.78

**Table 3:** Contact angles of the dense membranes.

Membrane	Contact Angle, °
PVA^dense^	67 ± 1
PVA-0-20^dense^	66 ± 1
PVA-1-20^dense^	65 ± 1
PVA-3-20^dense^	60 ± 2
PVA-5-20^dense^	41 ± 3

**Table 4:** Swelling characteristics of the dense membranes in water.

Dense Membranes	Swelling Degree, %
PVA^dense^	128 ± 3
PVA-0-20^dense^	125 ± 2
PVA-1-20^dense^	124 ± 1
PVA-3-20^dense^	123 ± 2
PVA-5-20^dense^	115 ± 1

**Table 5:** Transport properties of the supported membranes prepared using bulk modifications.

Membrane	Permeation Flux, kg/(m^2^h)	Water Content in Permeate, wt %
PVA-0-20	0.233	94.5
PVA-5-20	0.241	96.8

**Table 6:** Transport properties of supported PVA-0-20 and PVA-5-20 membranes prepared using surface LbL modification after pervaporation of 80 wt % isopropanol–20 wt % water mixture at 22 °C.

Pair of PEL	Charge Concentration (Number of Ion Pairs/Number of C Atoms in Repeat Unit)	Number of Bilayers	Membranes			
			PVA-0-20		PVA-5-20	
			Permeation Flux, kg/(m^2^h)	Water Content in Permeate, wt %	Permeation Flux, kg/(m^2^h)	Water Content in Permeate, wt %
PSS, PAH	0.09	5 bilayers	0.250	92.0	0.282	95.5
		10 bilayers	0.270	90.6	0.296	92.6
PSS, CS	0.0625	5 bilayers	0.334	94.0	0.340	95.6
		10 bilayers	0.229	89.3	0.252	93.3

**Table 7:** Transport properties of supported PVA-5-20 membranes with two, five, and seven bilayers of PSS and CS after pervaporation of 80 wt % isopropanol–20 wt % water mixture at 22 °C.

Membrane	Permeation Flux, kg/(m^2^h)	Water Content in Permeate, wt %
PVA-5-20/LbL-2^PSS,CS^	0.248	96.2
PVA-5-20/LbL-5^PSS,CS^	0.340	95.6
PVA-5-20/LbL-7^PSS,CS^	0.341	93.1

**Table 8:** The transport properties after the pervaporation of 80 wt % isopropanol–20 wt % water mixture at 22 °C for PVA-5-20 membrane with the variation of LbL modification conditions.

Pair of PEL	Number of Bilayers	Permeation Flux, kg/(m^2^h)	Water Content in Permeate, wt %
PSS, CS	5.5	0.291	95.6
PAA, CS	5	0.267	89.6
